# Cytological and molecular characterization of carotenoid accumulation in normal and high-lycopene mutant oranges

**DOI:** 10.1038/s41598-017-00898-y

**Published:** 2017-04-10

**Authors:** Peng-jun Lu, Chun-yan Wang, Ting-ting Yin, Si-lin Zhong, Don Grierson, Kun-song Chen, Chang-jie Xu

**Affiliations:** 1 Zhejiang Provincial Key Laboratory of Horticultural Plant Integrative Biology, Zhejiang University, Zijingang Campus, Hangzhou, 310058 China; 2 State Key Laboratory of Agrobiotechnology, the School of Life Sciences, The Chinese University of Hong Kong, Hong Kong, China; 3 Division of Plant and Crop Sciences, School of Biosciences, University of Nottingham, Sutton Bonington Campus, Sutton Bonington, LE12 5RD UK

## Abstract

Ripe Cara Cara sweet orange contains 25 times as much carotenoids in flesh as Newhall sweet orange, due to high accumulation of carotenes, mainly phytoene, lycopene and phytofluene. Only yellow globular chromoplasts were observed in Newhall flesh. Distinct yellow globular and red elongated crystalline chromoplasts were found in Cara Cara but only one type of chromoplast was present in each cell. The red crystalline chromoplasts contained lycopene as a dominant carotenoid and were associated with characteristic carotenoid sequestering structures. The increased accumulation of linear carotenes in Cara Cara is not explained by differences in expression of all 18 carotenogenic genes or gene family members examined, or sequence or abundance of mRNAs from phytoene synthase (*PSY*) and chromoplast-specific lycopene β-cyclase (*CYCB*) alleles. 2-(4-Chlorophenylthio)-triethylamine hydrochloride (CPTA) enhanced lycopene accumulation and induced occurrence of red crystalline chromoplasts in cultured Newhall juice vesicles, indicating that carotenoid synthesis and accumulation can directly affect chromoplast differentiation and structure. Norflurazon (NFZ) treatment resulted in high accumulation of phytoene and phytofluene in both oranges, and the biosynthetic activity upstream of phytoene desaturase was similar in Newhall and Cara Cara. Possible mechanisms for high carotene accumulation and unique development of red crystalline chromoplasts in Cara Cara are discussed.

## Introduction

Carotenoid pigments are a large family of isoprenoid compounds essential for plant growth and development. They play key roles in harvesting light for photosynthesis, protecting plants from high light stress, providing flowers and fruits with bright yellow, red, or orange colors for attracting animals and thus facilitate pollination and seed dispersal, as well as serving as precursors for biosynthesis of ABA, strigolactones, and some volatiles involved in other interactions between plants and animals^1, 2^. Carotenoids are also important compounds for human health, supply a significant antioxidant function, and protect humans from cardiovascular diseases and carcinogenesis^3–5^.

Carotenoids include carotenes, which lack oxygen atoms, and xanthophylls which contain oxygen (Supplementary Fig. S1). The main backbone of the pathway for biosynthesis of carotenoids involves over 20 enzymes, including 1-deoxy-D-xylulose 5-phosphate synthase (DXS), the first enzyme for the 2-C-methyl-D-erythritol 4-phosphate (MEP) pathway, phytoene synthase (PSY), the enzyme for production of the first carotenoid molecule phytoene, phytoene desaturase (PDS), the subsequent enzyme for desaturation of phytoene, and three lycopene cyclases for production of cyclic carotenes^6–8^ (Supplementary Fig. S1). The pathway has several critical controlling points, identified through application of inhibitors, such as a PDS inhibitor norflurazon (NFZ) and a lycopene cyclase inhibitor 2-(4-chlorophenylthio)-triethylamine hydrochloride (CPTA)^9, 10^ (Supplementary Fig. S1), and *via* gene expression analysis and genetic transformation, or by analyzing various mutants or natural varieties with contrasting carotenoid accumulation^1, 3^. However, while great variations exist in content and composition of carotenoids among species, and even varieties, there are many aspects of their biosynthesis and regulation that are still poorly understood^11–13^.

Various mechanisms have been reported for the differential accumulation of carotenoids among different varieties. Mutation in structural gene sequences is one of the most common explanations. For example, *r*, *Delta*, *Beta*, *old-gold* (*og*) and *old-gold crimson* (*og* ^*c*^), and *tangerine* tomatoes all result from carotenogenic gene mutation^14–17^. In watermelon, a critical single nucleotide polymorphism (SNP) in the lycopene β-cyclase gene (*LCYB*) determines the segregation of flesh color, either canary yellow or red, in tested cultivars^18^. In loquat, our previous study suggested that a deletion in phytoene synthase member 2A (*PSY2A*) was responsible for the lack of carotenoids in white-fleshed tissues^19^. Changes in gene expression can also be involved in differential accumulation of carotenoids. In carrot root, the difference in accumulation of carotenoids in red and yellow cultivars can be partially explained by the variations in expression of biosynthetic genes^20^. In some cases, both nonsense mutation and loss of expression of a gene can be involved, as reported for the capsanthin/capsorubin synthase gene (*CCS*) in two yellow pepper cultivars^11^.

Apart from sequence mutation or altered expression of carotenoid biosynthetic genes, a number of studies have shown that carotenoid accumulation was also affected indirectly by chromoplast development. The presence of only trace amounts of carotenoids in a white carrot has been demonstrated to be the result of significantly fewer chromoplasts^21^. Similarly, as revealed in our previous studies, the failure to develop normal chromoplasts in flesh tissue of a white-fleshed loquat is associated with the lack of carotenoid accumulation^22^. On the other hand, enhanced accumulation of carotenoids is often accompanied by enhanced plastid biogenesis, as in *high pigment* (*hp*) tomatoes, where the enhanced accumulation of carotenoids has been found to be related to increased number or size of chromoplasts^1, 21^. For *Orange* (*Or*) cauliflower mutants, high accumulation of β-carotene was explained by enhanced conversion from proplastids and other colourless plastids to chromoplasts resembling flattened sheets^23, 24^.

Accumulation of carotenoids in mature citrus fruits also varies among species and cultivars^25^ and some mutants have been reported with remarkable changes in carotenoid accumulation. Pinalate orange, a yellow mutant of Navelate orange, accumulates linear carotenoids in the flavedo to 90% of total carotenoids, much higher than the 2% for Navelate, and it was suggested that a defect in ζ-carotene desaturase (*ZDS*) or *ZDS*-associated factors may be the underlying reason^26^. Rohde Red Valencia orange, a mutant of Valencia orange with a deeper color, accumulates a higher level of β-cryptoxanthin and violaxanthin which was suggested to be related to higher expression of *PDS*, *ZDS* and zeaxanthin epoxidase (*ZEP*)^27^. Lycopene-accumulating mutants have been reported as early as the 1930s in several species including sweet oranges, grapefruits and pummelos^28^. Recently, the possible mechanisms involved in the lycopene accumulation phenotype have been investigated by several workers, using phytochemical, genetical, metabolomic, transcriptomic and proteomic approaches^28–34^. In Star Ruby grapefruit, preferential expression of a non-functional allele of *β-LCY2*, a synonym for *chromoplast-specific lycopene β-cyclase* (*CYCB*), was proposed to be an additional regulatory mechanism responsible for accumulation of high amounts of lycopene^32^. However, further study on the white fleshed Marsh grapefruit and red fleshed Star Ruby grapefruit showed the alleles with high (*β-LCY2a*) and low (*β-LCY2b*) activity were expressed in similar proportions^35^. In the red-flesh Hong Anliu sweet orange mutant, significant changes in expression of numerous genes, the amount of various primary and secondary metabolites, as well as abundance of some plastid-localized proteins were observed, compared to wild type^31, 33, 34^.

Profiles of carotenoid accumulation in mature fruit of Cara Cara, a red-fleshed sweet orange mutant accumulating lycopene, have been characterized previously^28, 36^, but the underlying explanation for the greater carotene content has not been elucidated. In this study, the mechanisms for accumulation of high amounts of carotenes were explored by studying chromoplast structure, expression of carotenogenic genes, and the effects of biosynthetic inhibitors. The presence of an extra type of chromoplast that accumulates lycopene in some flesh cells explains the increased carotenoids in Cara Cara, which is of general interest for plastid differentiation and carotenoid biosynthesis research. Addition of the lycopene cyclase inhibitor CPTA caused cultured juice vesicles of Newhall, an ordinary sweet orange, to increase carotenoid content and produce red crystalline chromoplasts, indicating carotenoid accumulation can lead to differentiation of red chromoplast. Results of norflurazon treatment, however, suggested that massive accumulation of carotenoids in Cara Cara did not result from enhanced biosynthesis upstream of phytoene.

## Results

### Fruit color changes in Newhall and Cara Cara oranges during development

**Figure 1:**
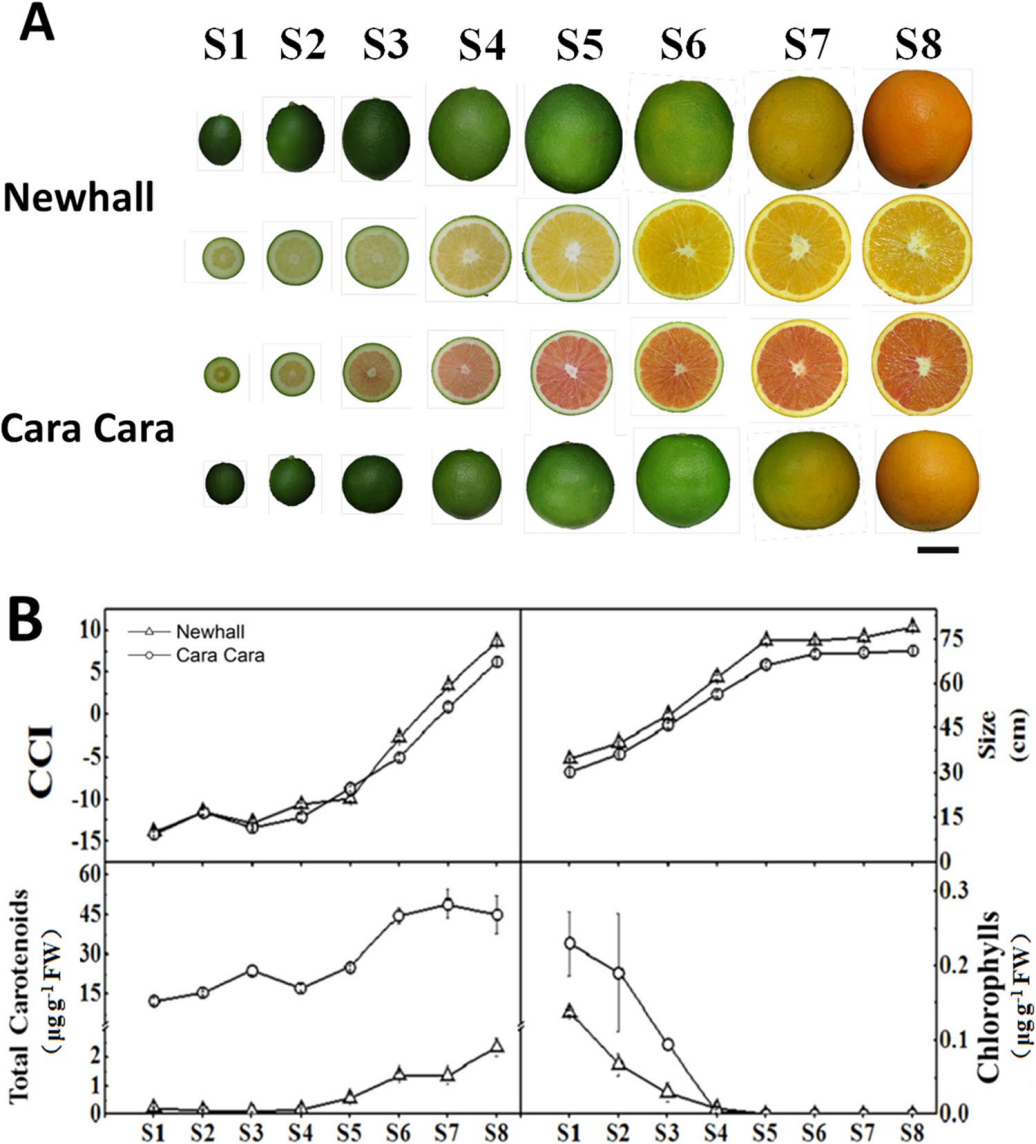
Fruit shape, size, color, carotenoids and chlorophylls in Newhall and Cara Cara oranges. (**A**) External and internal colors of Newhall and Cara Cara fruits during development and ripening at different stages (S1–S8). Bar, 2 cm. (**B**) Changes in peel CCI (citrus colour index), fruit size (transverse diameter), content of flesh total carotenoids and flesh chlorophylls during fruit development and ripening in Newhall and Cara Cara oranges. The bars represent SE from three biological replicates.

Fruit growth and peel color changes were similar for both Newhall and Cara Cara oranges, with both size and citrus color index (CCI) showing an increasing trend during fruit development and maturation (Fig. 1). CCI of fruit peel changed from negative to positive, corresponding with the color change from green to yellow or orange-yellow. There were large differences in flesh color between two cultivars during fruit development, with ripe Cara Cara fruit being red and Newhall yellow (Fig. 1A).

### Pigment changes in flesh of Newhall and Cara Cara oranges during development

**Figure 2:**
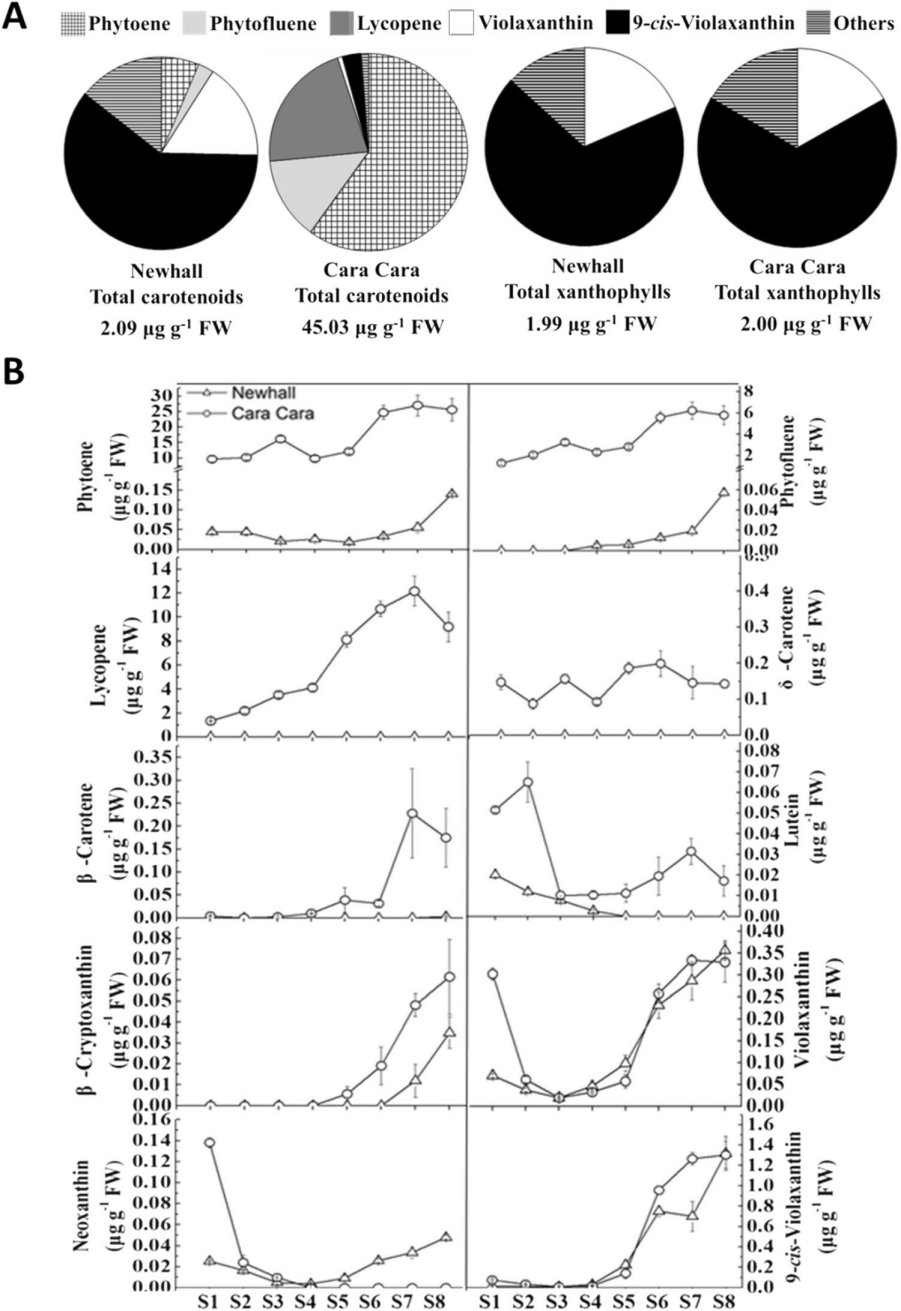
Profiles of carotenoids in Newhall and Cara Cara oranges. (**A**) Composition and amount of total carotenoids or total xanthophylls in flesh of Newhall and Cara Cara ripe fruit. (**B**) Changes in content of carotenoids in flesh of Newhall and Cara Cara oranges during fruit development and ripening. The bars represent SE from three biological replicates.

The flesh colour pattern was ascribed to differences in content and composition of chlorophylls and carotenoids. Both cultivars accumulated a small quantity of chlorophylls at the early stages, which decreased gradually until S5 (Fig. 1B). In Newhall, only trace amount of carotenoids were present until S5 and then increased steadily during fruit maturation. However, in Cara Cara, large amount of carotenoids accumulated in flesh tissues even at S1, and then increased to around three fold during fruit maturation (Fig. 1B). During the whole development and maturation stages, carotenoids in flesh of Cara Cara were between 19 and 56 times higher than in Newhall (Fig. 1B). This increment resulted mainly from enhanced accumulation of carotenes, but, taking S8 fruit as an example, the amount of total xanthophylls in the two sweet orange cultivars was similar (Fig. 2A).

There were major differences in the composition of carotenoids in flesh in the two sweet orange cultivars. In Newhall ripe fruit, the three most abundant carotenoids were all xanthophylls, 9-*cis*-violaxanthin, violaxanthin, and luteoxanthin, while in Cara Cara the linear carotenes, phytoene, lycopene, and phytofluene were the most abundant (Fig. 2A). These differences between two cultivars were also observed at other developmental stages (Fig. 2B).

### Light microscopy of chromoplasts in flesh cells of Newhall and Cara Cara oranges during fruit development

**Figure 3:**
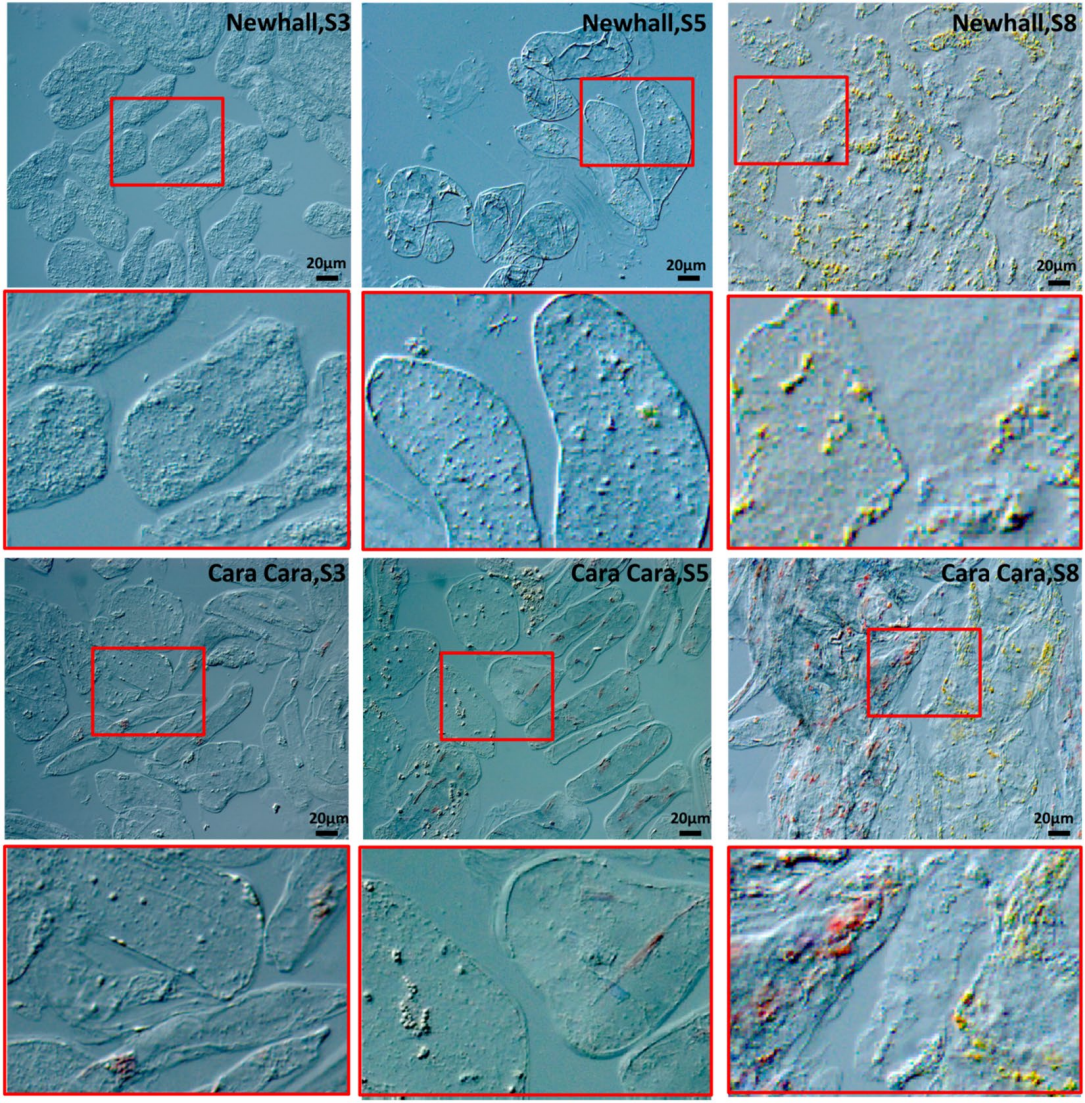
Cells of Newhall and Cara Cara orange flesh at early stage S3, middle stage S5, ripe stage S8 under DIC (differential interference contrast) microscopy. Bar, 20 μm.

A difference in chromoplast development was observed for the two cultivars. Only yellow globular chromoplasts were observed in all flesh cells of mature Newhall fruit, while two distinct types, yellow globular ones and red crystalline ones, were found in different flesh cells of Cara Cara (Fig. 3). Crucially, for any individual cell, only one type of chromoplast was present and cells contained exclusively either red chromoplasts or yellow ones, often with cells containing each type found adjacent to each other (Fig. 3). A single cell with both types of chromoplasts was not observed, and this precluded the possibility of transition between two types of chromoplasts.

During fruit development, red crystalline chromoplasts appeared before the yellow globular ones. As shown in Fig. 3, in flesh tissue of Cara Cara fruit at S3, some cells harbored red crystalline chromoplasts while the others were devoid of chromoplasts, while at this stage no chromoplast could be observed in flesh cells of Newhall. At later stages, individual flesh cells contained substantial numbers of either yellow or red chromoplasts for Cara Cara and only yellow chromoplasts for Newhall (Fig. 3).

**Figure 4:**
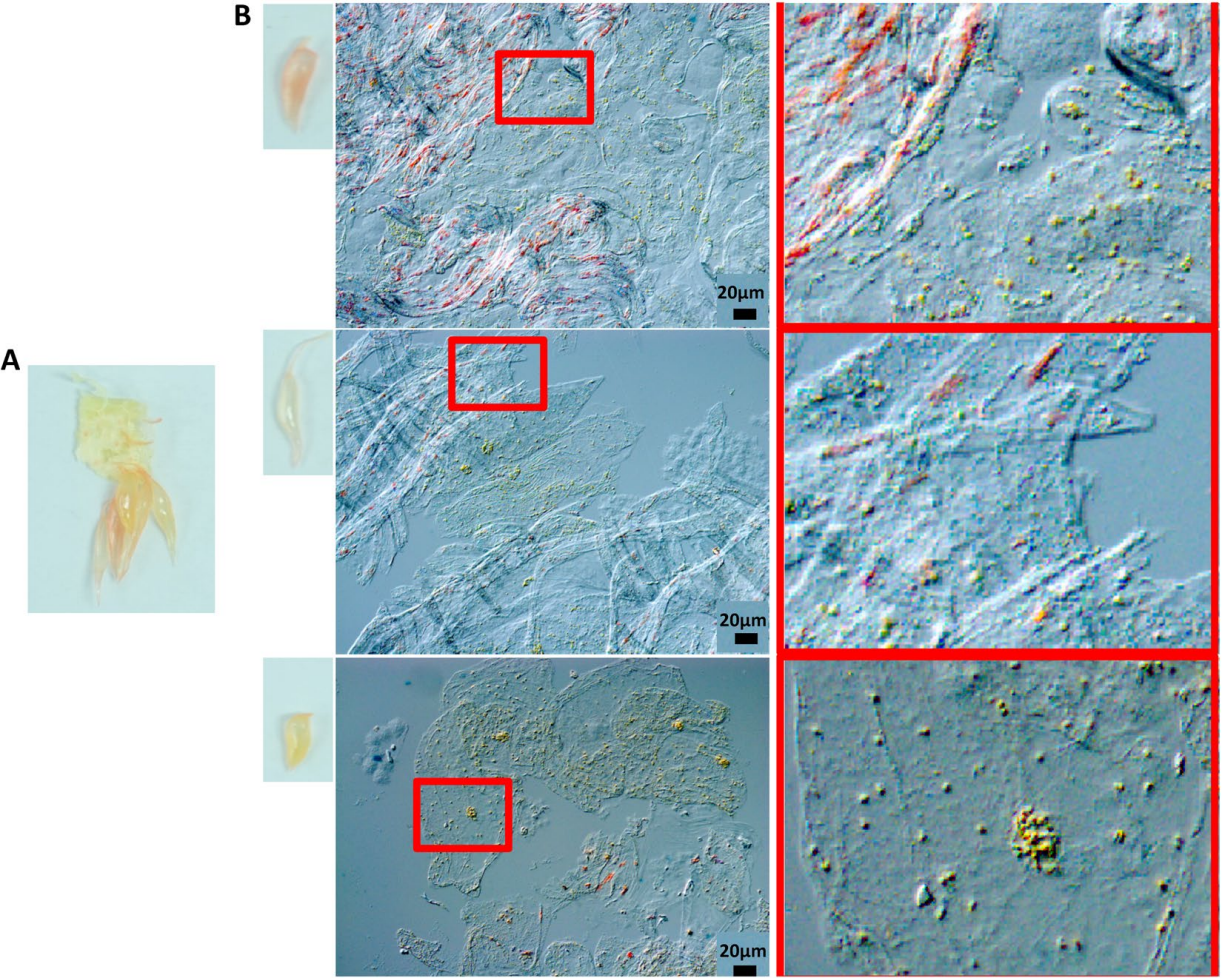
Microscopic observation of cells from different colored juice vesicles of Cara Cara ripe fruit. (**A**) Photograph of dissected Cara Cara fruit with different colored juice vesicles located close to each other. (**B**) Cells from red to yellow juice vesicles under DIC (differential interference contrast) microscope.

Despite the fact that Cara Cara flesh appeared to have a uniform red color in cross section (Fig. 1), a close examination showed that juice vesicles of different colours, from yellow to red, could be found under the microscope, even from neighboring sites (Fig. 4A). However, inside each vesicle, which contains multiple cells, two types of cells with distinct chromoplasts could still be found, no matter what the colour of the vesicle was (Fig. 4B). The color difference between these three groups of vesicles resulted from the different percentage, around 60%, 30% and 10% respectively, of cells with red chromoplasts.

### *In situ* analysis of carotenoids in flesh chromoplasts of Newhall and Cara Cara oranges by Raman microspectroscopy

**Figure 5:**
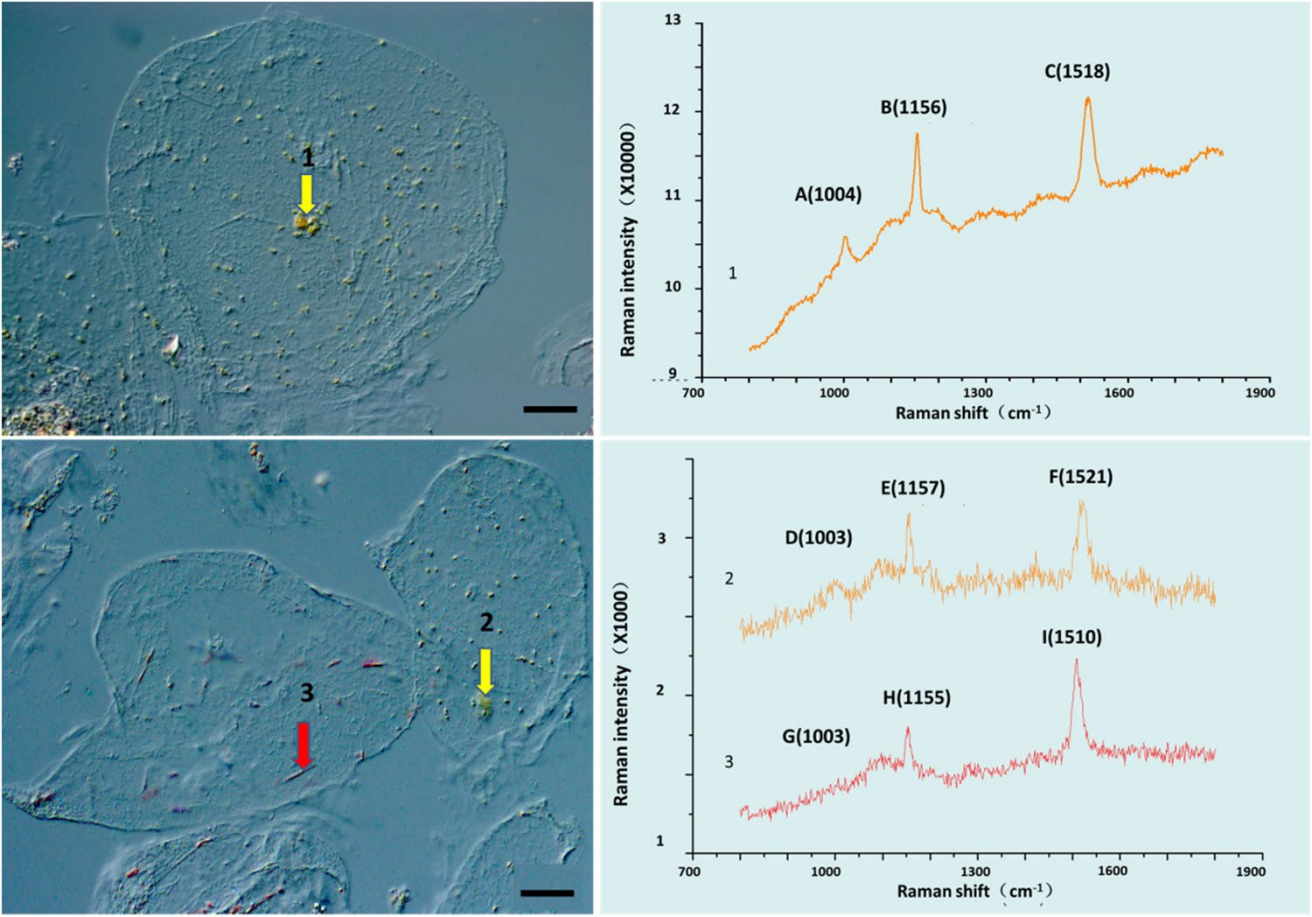
Raman microscope spectra of yellow globular chromoplast in Newhall (1), yellow globular chromoplast in Cara Cara (2), red crystalline chromoplast in Cara Cara (3). Bar, 50 μm. Line 1, 2 represent carotenoids with 9 carbon-carbon double bond; Line 3 represent lycopene with unique 11 carbon-carbon double bond.

It was technically not feasible to collect sufficient protoplasts harboring either type of chromoplasts for carotenoid analysis by HPLC. However, by *in situ* Raman confocal microscopy, we were able to establish that the red crystalline chromoplasts contain lycopene as a dominant carotenoid, as manifested by the presence of three peaks around 1510 cm^−1^, 1156 cm^−1^ and 1004 cm^−1^, consistent with the spectrum produced by tomato lycopene^37^, while the globular ones, either from Cara Cara or Newhall, did not (Fig. 5). The spectrums for globular chromoplasts from Cara Cara and Newhall were similar (Fig. 5), probably indicating other similarities in carotenoid composition.

### Ultrastructure of plastids in Newhall and Cara Cara flesh cells during fruit development

**Figure 6:**
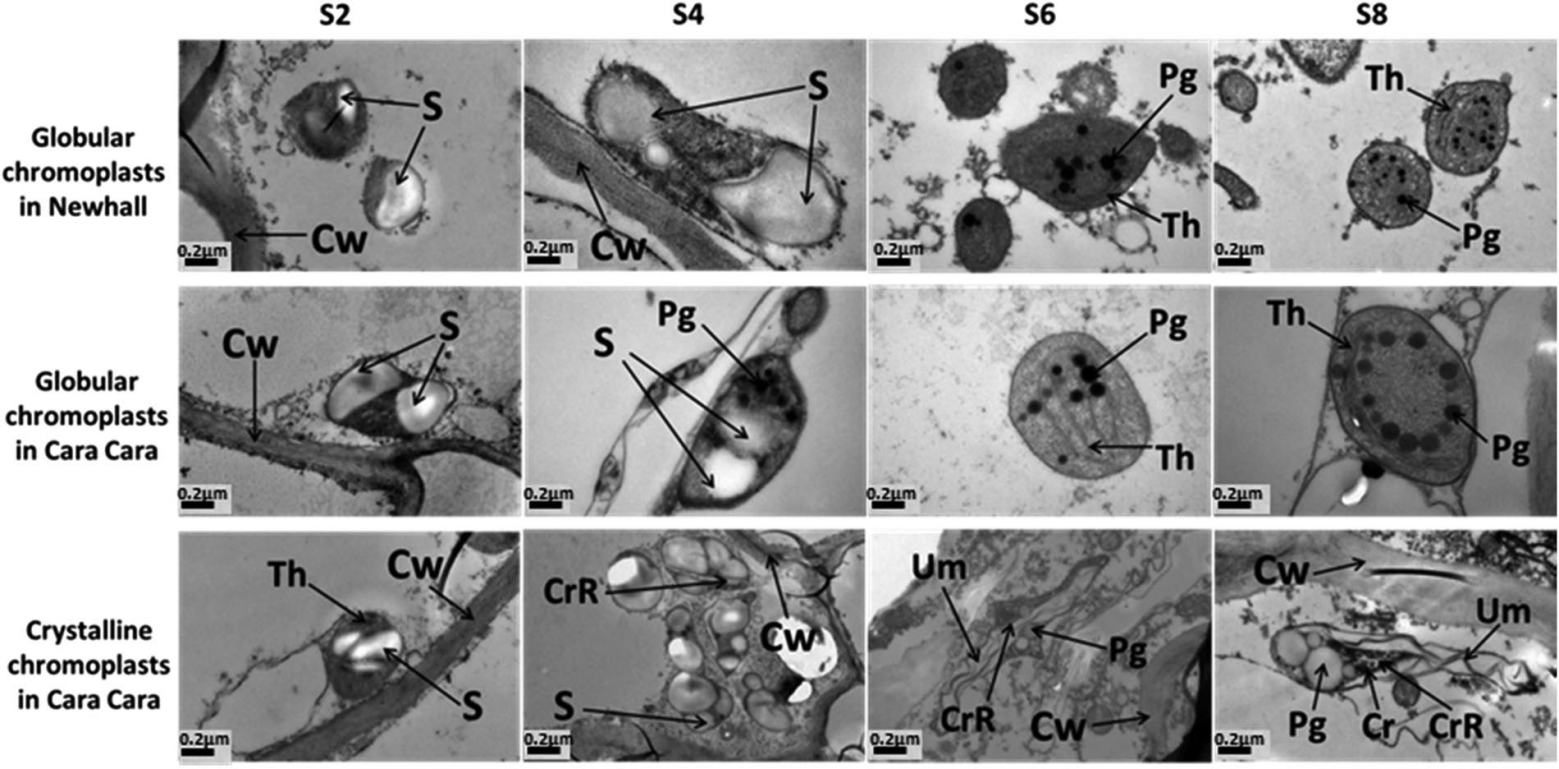
Ultrastructure of yellow globular chromoplast in Newhall, and yellow globular and red crystalline chromoplasts in Cara Cara viewed in the transmission electron microscope. Cr, crystal; CrR, crystalloid remnant; Cw, cell wall; Pg, plastoglobuli; S, starch; Th, thylakoid; UM, undulating membrane.

Differences in ultrastructure of plastids in flesh cells of the two cultivars were observed by TEM. During early stages (S2 and S4), the plastids contain starch granules and thylakoid structures (Fig. 6). At later stages (S6 and S8), consistent with the data from microscopy observation, only globular chromoplasts were found in Newhall flesh cells while both globular and crystalline ones were found in Cara Cara but in different flesh cells. The globular ones in Newhall and Cara Cara shared similar ultrastructure, containing thylakoid membranes and plastoglobules, while crystalline chromoplasts, present in Cara Cara only, had a distinct ultrastructure, characterized by the presence of large plastoglobules, crystals, crystal remnants and undulating membranes (Fig. 6).

### Sequences of *PSY1*, *LCYB* and *CYCB* in Newhall and Cara Cara

Since Cara Cara possesses high amount of carotenes including lycopene, here the sequences of *PSY1*, *LCYB* and *CYCB* were checked for possible mutation, since it has been established in other studies that these are potential explanations for differences in carotenoid content. For each gene, two highly homologous sequences were obtained. This is consistent with the fact that orange is a diploid plant with heterogeneous genetic background.

Sequencing *PSY1* cDNA revealed the existence of two alleles, named *PSY1-a* and *PSY1-b*, in both oranges (Supplementary Fig. S2). Twelve SNPs and an indel of 6 bp, resulting from of gain or loss of two repeats of TAA, were found when comparing two alleles (Supplementary Fig. S2). However, no sequence difference was observed between two orange cultivars, i.e., the *PSY1-a* sequences for both cultivars are identical and this is also true for the *PSY1-b* sequences (Supplementary Fig. S2). Sequence BLASTing of both alleles in NCBI (http://blast.ncbi.nlm.nih.gov/Blast.cgi) revealed that *PSY1-a* was identical to *PSY* in *C*. *unshiu*, while *PSY1-b* was identical to *C*. *maxima*. This is consistent with the result of Velasco and Licciardello (2014)^38^ who suggested that sweet orange arose from natural crosses between mandarin and pummelo.

Similarly, two alleles for *LCYB* and *CYCB* were identified from Newhall and Cara Cara. While 16 SNPs were found between LCYB alleles and 26 SNPs in *CYCB* alleles, no sequence difference was found between two cultivars (Supplementary Figs S3 and S4). The sequence of the *CYCB-a* allele was identical with *β-LCY2a*, *i.e*., allele a of *β-LCY2*, a synonym for *CYCB*, in Star Ruby grapefruit^32^ while *CYCB-b* had a synonymous SNP difference when compared with *β-LCY2b* ^32^.

### Carotenogenic gene mRNA levels in flesh of Newhall and Cara Cara oranges

**Figure 7:**
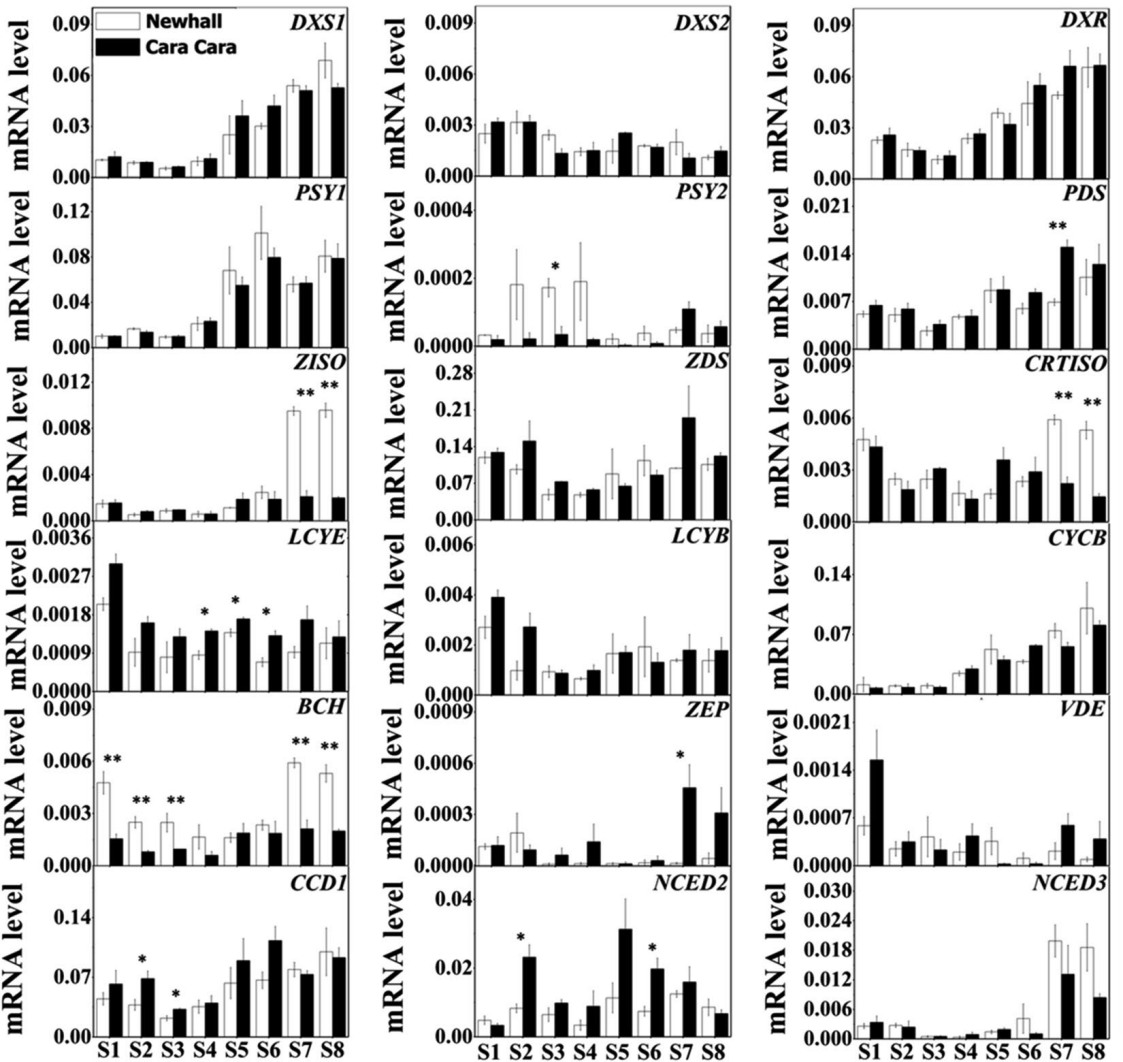
Expression of carotenogenic genes in flesh of Newhall and Cara Cara oranges during fruit development and ripening. The bars represent SE from three biological replicates_._ Asterisks indicate significant differences, P < 0.05 and P < 0.01 are designated by * and **, respectively. The abbreviation for the genes is the same as listed in Fig. 1.

The mRNA levels for carotenogenic genes in flesh of Newhall and Cara Cara oranges were compared (Fig. 7). Two members of *DXS* were expressed and the *DXS1* transcript accounted for over 95% of total *DXS* transcripts in both orange cultivars, and this was also true for *PSY* (Fig. 7). Transcript levels for *DXS1* and *PSY1* increased during fruit development, but no significant difference in transcript abundance of *DXS1* and *PSY1* was observed between two cultivars. Expression of genes involved in serial conversion of phytoene into lycopene was similar for most samples between the two cultivars, except for higher transcript level of *carotene isomerase* (*CRTISO*) and *ζ-carotene isomerase* (*ZISO*) in Newhall at the last two stages of fruit development. However, during the early stages, despite the existence of differences in carotenoid accumulation, the transcript abundance of *CRTISO* and *ZISO* was similar in the two cultivars. Expression of both *LCYB* and *lycopene ε-cyclase* (*LCYE*) was over 20 fold lower than that of *CYCB*, and the transcript level of *CYCB* was similar in both oranges. Transcript levels of *carotene β-ring hydroxylase* (*BCH*) were lower and that of *ZEP* higher in Cara Cara at some stages but not others. Furthermore, the transcript level of three carotenoid cleavage genes, *carotenoid cleavage dioxygenase* gene family member 1 (*CCD1*), *nine-cis-epoxycarotenoid dioxygenase* gene family members 2 and 3 (*NCED2*, *NCED3*), were similar overall in the two cultivars.

**Figure 8:**
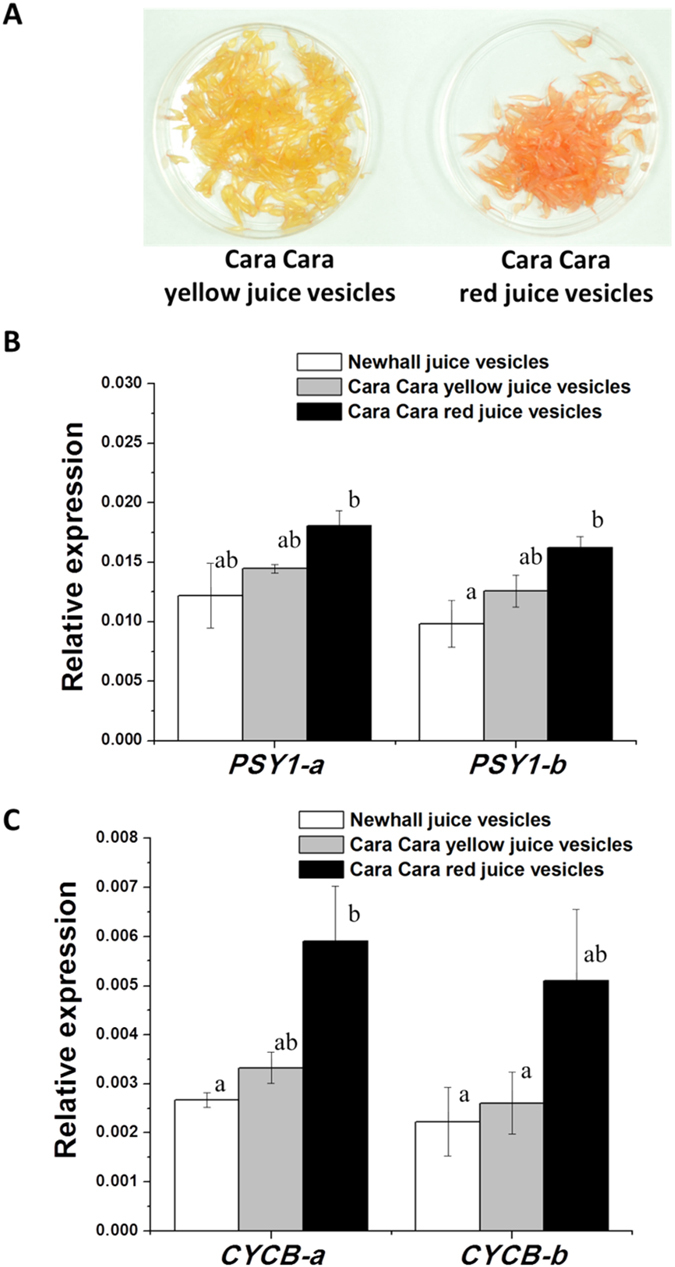
Expression of *PSY1* and *CYCB* alleles in different colored juice vesicles at S6. (**A**) Different color of yellow and red juice vesicles from the same Cara Cara fruit. (**B**) Expression level of two alleles of *PSY1* in Newhall juice vesicles and Cara Cara yellow, red juice vesicles. (**C**) Expression level of two alleles of *CYCB* in Newhall juice vesicles and Cara Cara yellow, red juice vesicles. The expression level of each of the *PSY1* and *CYCB* allele was measured by quantitative real-time PCR using allele-specific PCR primers (Supplementary Table S1). The bars represent SE from three biological replicates. A significance test was conducted and values with the same letter indicates no significant difference at P < 0.05.

The transcript levels for each of the *PSY1* and *CYCB* allele in yellow and red juice vesicles separated from the same fruit of Cara Cara (Fig. 8A) were analysed by quantitative real-time PCR using allele-specific PCR primers (Supplementary Table S1), and it was found that the expression of both *PSY1* and *CYCB* alleles was slightly higher in Cara Cara red juice vesicles than in Cara Cara yellow vesicles and Newhall vesicles, but no obvious bias in expression between alleles was observed (Fig. 8B).

### Effects of NFZ and CPTA treatments on *in vitro* cultured flesh tissues

**Figure 9:**
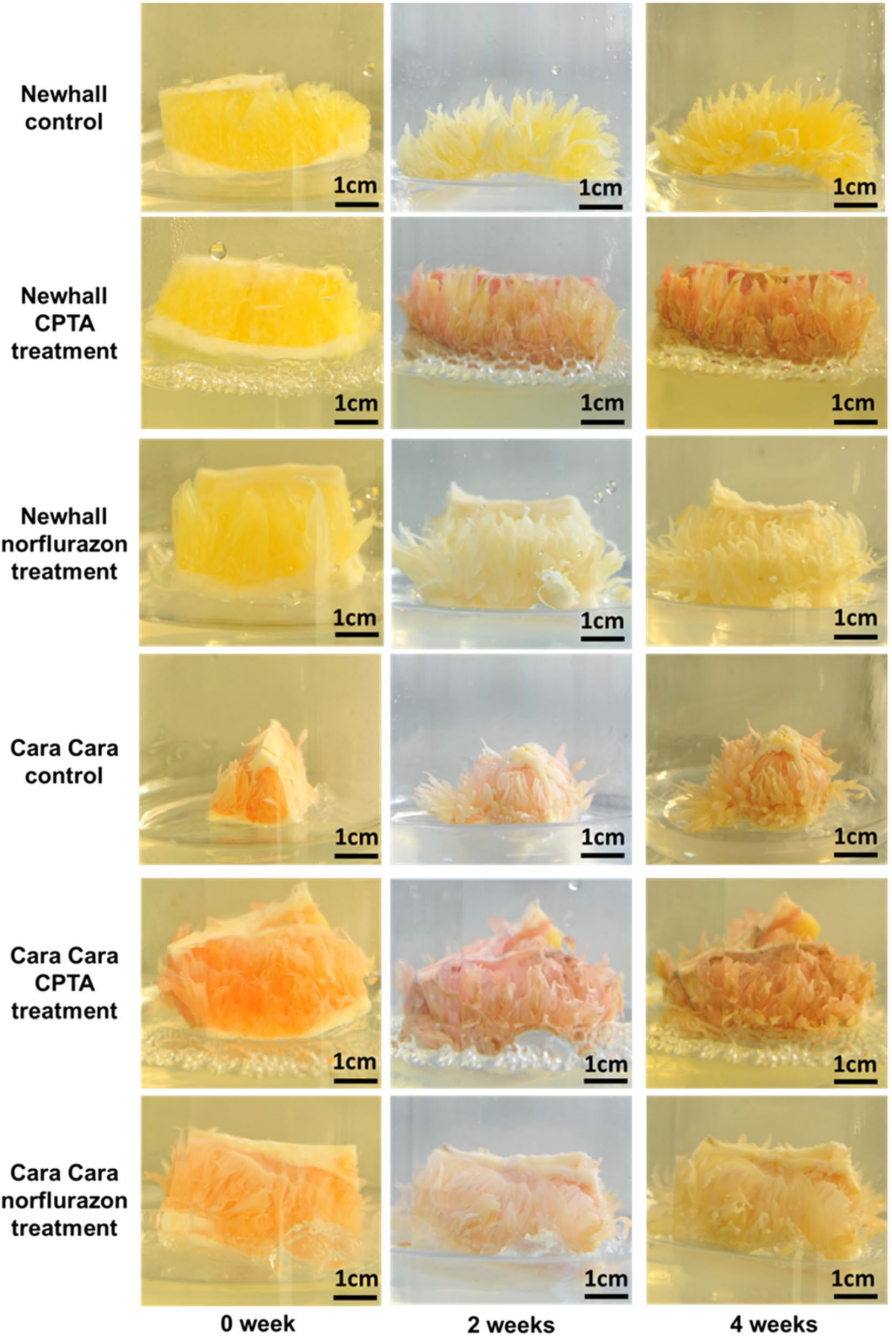
*In vitro* cultured flesh tissues of Newhall and Cara Cara following CPTA and norflurazon treatments. The 0 week tissues were separated from fruit at the S5 stage. CPTA, 2-(4-chlorophenylthio)-triethylamine hydrochloride.

**Figure 10:**
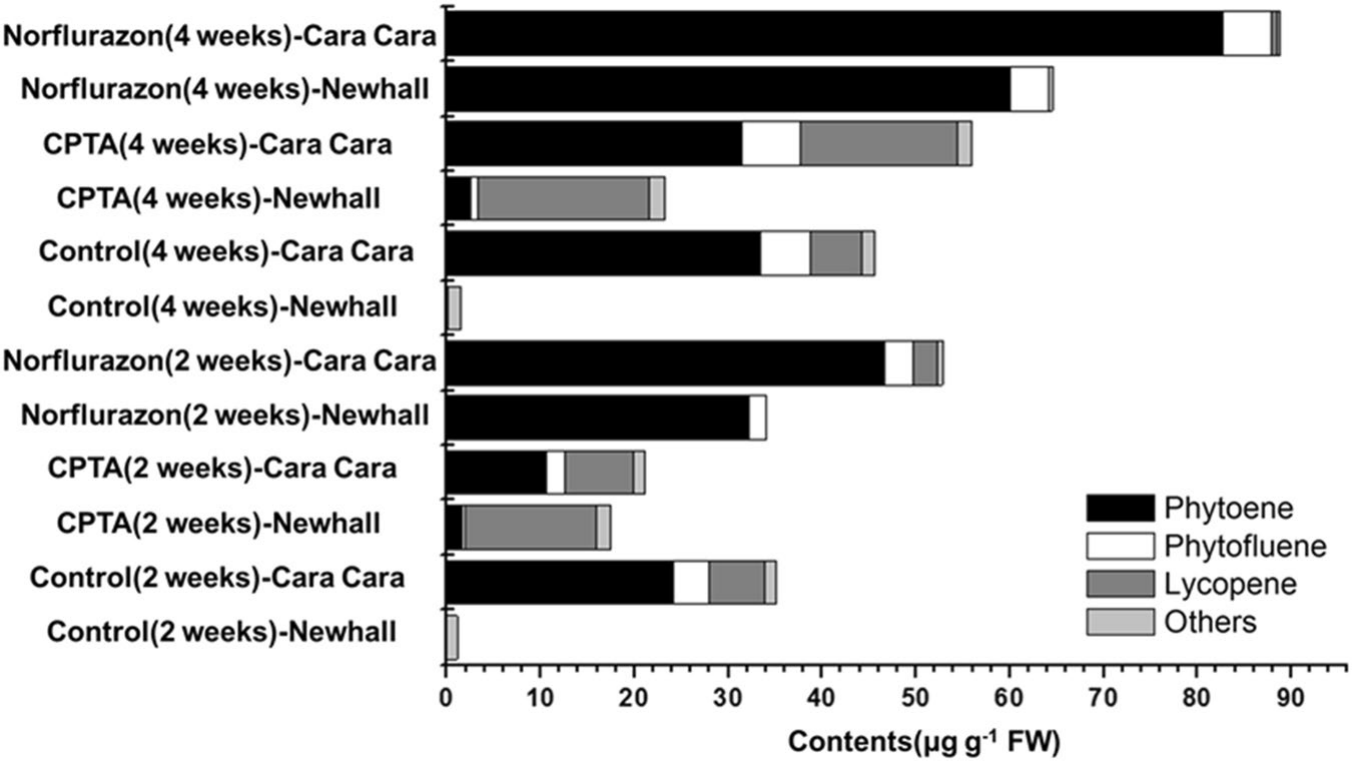
Amount and composition of carotenoids in *in vitro* cultured flesh tissues of Newhall and Cara Cara following CPTA and norflurazon treatments. The 0 week tissues were separated from fruit at the S5 stage. CPTA, 2-(4-chlorophenylthio)-triethylamine hydrochloride.

As expected, NFZ treatment induced an albino phenotype in both Newhall and Cara Cara flesh tissues (Fig. 9) and, although the amount of total carotenoids increased, colorless carotenoids phytoene and phytofluene were the predominant ones, accounting for over 90% of total carotenoids for both oranges at four weeks following treatment (Fig. 10). The increment in total carotenoids between treated flesh tissues and control following 4-week NFZ treatment was 63.06 μg g^−1^FW in Newhall and 43.18 μg g^−1^FW in Cara Cara (Supplementary Table S2).

For both oranges, flesh tissues became deeper red following CPTA treatment during 4-weeks in culture (Fig. 9). The red pigment in CPTA-treated flesh tissue was mainly lycopene. In Newhall, lycopene accumulated up to 79% and 78% of total carotenoids following 2- and 4-week CPTA treatment, respectively, and accumulation of small amounts of phytoene and phytofluene was also observed (Fig. 10). In contrast, control Newhall flesh tissues did not contain phytofluene and lycopene, and only trace amounts of phytoene. In Cara Cara, CPTA treatment also raised the percentage of lycopene among total carotenoids; however, phytoene was still the most predominant carotenoid (Fig. 10). At four weeks following CPTA treatment, the increment in total carotenoids between treated flesh tissues and control was 21.72 μg g^−1^FW in Newhall and 10.31 μg g^−1^FW in Cara Cara (Supplementary Table S2).

**Figure 11:**
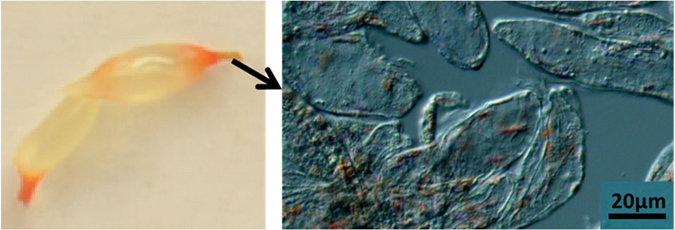
Occurrence of red crystalline chromoplasts in cells from CPTA treated Newhall flesh tissues. The juice vesicle was from flesh tissues separated from fruit at the S5 stage and then treated with CPTA for four weeks. CPTA, 2-(4-chlorophenylthio)-triethylamine hydrochloride.

The Newhall juice vesicles following 4-week CPTA treatment were taken for microscopic analysis, and it was found that red crystalline chromoplasts occurred, especially in the deeper red part (Fig. 11). In addition, the CPTA treated Newhall albedo tissue was also checked and red crystalline chromoplasts were found in all cells (Supplementary Fig. S5). However, no colored plastids were observed in control Newhall albedo layers. In Cara Cara control albedo layers (without CPTA), red crystalline chromoplasts could also be observed in some cells (Supplementary Fig. S5).

## Discussion

Differences in carotenoid accumulation, varying in some cases up to hundreds of fold in total carotenoid content, have been reported between wild type and mutants, as well as among cultivars, in various plants^11, 18, 19, 39–41^. The tremendous differences often resulted from no or trace accumulation of carotenoids in a mutant or cultivar, such as the *r* tomato and white-fleshed loquats caused by the loss of function mutation of *SlPSY1* and *EjPSY2A* ^14, 19^, and Early Moonbeam watermelon^39^. Here the difference in carotenoid accumulation between two sweet orange cultivars is a different case, since the ordinary orange Newhall contains normal level of carotenoids while the mutant Cara Cara contains over 20 times more in flesh during fruit development. Higher-than-normal accumulation of carotenoids in mutant fruits has also been reported in *hp1*, *hp2* and *hp3* tomatoes as well as a red-fleshed papaya and Hong Anliu sweet orange, however, the increment in fruits of these mutants was moderate, not exceeding six fold^12, 29, 42^.

Tremendously enhanced accumulation of carotenoids was reported in the curd of a cauliflower mutant *Or*, but the carotenoid accumulated was mainly β-carotene^43^, quite different from the linear carotenes in Cara Cara. Accumulation of lycopene in fruit of red-fleshed mutants rather than wild-type or ordinary cultivars has been reported for several plants, including papaya and citrus^12, 28, 29, 32^. However, the carotenoid composition in Cara Cara fruit is unique among these lycopene-accumulating mutants, characterized by accumulation of higher amounts of another two other linear carotenes, phytoene, which was twice the amount of lycopene, and phytofluene, as well as normal amounts of xanthophylls (Fig. 2A). This contrasts with the results for ordinary tomato and red-fleshed watermelon, where the amount of phytoene is normally less than 10% of lycopene^44, 45^, for red-fleshed grapefruits, which contains high amount of phytoene and phytofluene but the xanthophyll amount is quite low^28^, and for red-fleshed Hong Anliu sweet orange, which does not contain phytoene in flesh tissues^29^. Thus the unique carotenoid accumulation in Cara Cara may indicate involvement of an unusual mutation.

The possible cytological mechanisms for the unique carotenoid accumulation in Cara Cara were investigated. Carotenoids are synthesized and accumulated in plastids. There are several types of plastids in plants, and chromoplasts are the main plastid type for carotenoid accumulation in ripe fruit. Chromoplasts vary in their morphology and types of carotenoid-accumulating substructures and can be classified as globular, crystalline, membranous, fibrillar, tubular and vesicle types^46^. Here yellow and globular chromoplasts were found in both oranges but red and crystalline ones were only observed in Cara Cara (Fig. 3).

Chromoplast development may involve either transition from other plastids such as chloroplasts, amyloplasts, *etc*., or direct biogenesis from proplastids, and particular patterns of development are important for carotenoid accumulation in different wild type and mutant plants. In *hp1*, *hp2* and *hp3* tomatoes, the enhanced accumulation of carotenoids was related to increased chromoplast number and size^42^. The *Or* cauliflower mutant possesses a much higher ability for conversion of proplastids and other colorless plastids to yellow chromoplasts and accumulates high amounts of β-carotene^24^. Overexpression of the cauliflower *Or* in potato resulted in enhanced carotenoid accumulation in tubers and promoted the development of chromoplasts with similar ultrastructure to those in *Or* cauliflower^47^. Through Raman microscopy, we showed that the red crystalline chromoplasts in Cara Cara contain lycopene, while globular chromoplasts do not (Fig. 5). Therefore, it is clear that occurrence of red crystalline chromoplasts is closely related to the accumulation of lycopene, and probably other linear carotenes as well. Association of the occurrence of crystalline chromoplasts with lycopene accumulation has also been observed in red-fleshed papaya^12^. In peppers, it has also been shown that variations in shape, size and ultrastructure of chromoplasts are associated with differences in carotenoid amount and composition in different cultivars^48^.

Because of the close association between occurrence of red crystalline chromoplasts and the accumulation of carotenes in Cara Cara, it might be tempting to conclude that the development of this type of chromoplast determines the unique carotenoid accumulation in citrus. However, through CPTA treatment, it was shown that enhanced linear carotene accumulation can result in development of crystalline chromoplasts in ordinary orange flesh (Fig. 11), which suggests that chromoplast sink capacity is not the factor limiting the accumulation of carotenoids in ordinary citrus, and development of chromoplasts, even of a specific type, can be stimulated to adapt for the enhanced biosynthesis of carotenoids, and, in this case lycopene accumulation caused crystalline chromoplasts to develop where they normally do not occur. Perturbation of plastid development by modified carotenoid synthesis has also been reported in other plants. In tomato overexpressing *SlPSY1*, chromoplast-like structures developed prematurely during fruit development^49^, and in maize, it was found that enhanced carotenoid biosynthesis through modification of natural PSY1 to increase enzyme activity resulted in distorted plastid shape and formation of fibrillar plastoglobulin^50^.

In summary, the causative relationship between chromoplast development and carotenoid accumulation is complicated, and may not be the same in different cases. In ordinary orange, accumulation of carotenoids might be limited by metabolism rather than chromoplast development, and in Cara Cara, occurrence of red crystalline chromoplasts might be not the reason for, but the result of enhanced carotenoid accumulation.

Several possible metabolic control points for enhanced carotenoid accumulation in Cara Cara were evaluated. PSY is the enzyme which catalyses production of the first carotenoid molecule phytoene, and is implicated as a major metabolic control point for carotenoid synthesis^49^. Therefore, it is important to evaluate whether PSY is involved in higher carotenoid accumulation in Cara Cara. Three PSY members are present in the citrus genome, however, in flesh tissues, only *PSY1* is strongly expressed, accounting for over 95% of total *PSY* transcripts in both orange cultivars, while the expression of *PSY2* is quite weak (Fig. 7 of this study; Peng *et al*.^51^) and *PSY3* transcripts are undetectable (data not shown; Peng *et al*.^51^). Evolutionarily, oranges are offsprings from several natural crossing between pummelo and mandarin^52^. Therefore, oranges are heterozygous genetically. Here we observed two alleles for *PSY1*, with over 98% nucleotide homology between alleles, and there was no difference in sequence or allele expression between the two sweet orange cultivars in this study (Fig. 8; Supplementary Fig. S2). These data suggested that *PSY1* is not the gene responsible for high carotene accumulation in Cara Cara, in accordance with the suggestions made previously by Alquézar *et al*.^30^. Recently, posttranscriptional regulation of PSY activities was reported in *Arabidopsis* ^53^, however, this may not occur in Cara Cara, since the increment in phytoene accumulation during a 4-week incubation of juice vesicle tissues on NFZ medium was slightly lower (49.32 μg g^−1^FW) in Cara Cara than in Newhall (60.06 μg g^−1^FW) (Supplementary Table S2), indicating that PSY activity is not higher in Cara Cara.

Lycopene cyclase genes include *LCYB* and *LCYE*, predominantly expressed in green tissues, as well as *CYCB*, expressed specifically in chromoplast-containing tissues^16^. *CYCB* is the gene with strongest transcript abundance in flesh tissues of oranges, being around 100 times higher in amount than *LCYB* and *LCYE* mRNA (Fig. 7). Similar to the results for *PSY1*, two alleles of *CYCB* were found, but no difference was observed in either allele between the two cultivars, and no obvious bias in expression of alleles was found (Fig. 8), which is quite similar to the data obtained by Alquézar *et al*.^35^ for comparison between a white-fleshed and a red-fleshed grapefruit. Therefore, *CYCB* is unlikely to be the gene responsible for high carotene accumulation in Cara Cara. This conclusion was also supported by the data from CPTA treatment of cultured flesh tissues, where CPTA-treated Newhall flesh tissues contained much higher amounts of lycopene than phytoene, but did not have a carotenoid composition resembling the Cara Cara control (Fig. 10; Supplementary Table S2).

Though the carotenoid amount is about 25 times less in flesh of ripe Newhall fruit compared to Cara Cara, the Newhall flesh tissues can be induced to accumulate considerable amount of carotenoids by CPTA and NFZ (Fig. 10, Supplementary Table S2), implying that the upstream biosynthesis ability is not lower in Newhall. Therefore, it would be valuable to further evaluate whether the carotenoid degradation capacity is different between these two cultivars and if it is, to identify the gene responsible for the high carotenoid amount in Cara Cara. This is an important area for future study since in some other plant tissues, e.g., white-fleshed peach fruit and white-coloured chrysanthemum petals, enhanced carotenoid degradation activity has been reported to be a factor resulting in absence of carotenoid accumulation^40, 54^. Allelic expression analysis of key carotenoid catabolic genes should be an important focus for future study of the possible mechanism(s) and nature of the mutation responsible for the high carotene content of Cara Cara fruit.

Another interesting aspect of this study is observation of the occurrence of two different types of cells with distinct types of chromoplasts in Cara Cara flesh. To our knowledge, the presence of adjacent cells in the same tissue, each with an exclusive type of chromoplast, either globular or crystalline ones, but not both, has not been reported in plants previously.

Cara Cara is a mutant of ordinary orange^36^. The possibility that the mutation is in the plastid genome is not likely because in that case co-existence of both types of chromoplasts in a single Cara Cara cell would be expected. A remaining question would be how a mutation can result in the occurrence of two different types of cells with distinct type of chromoplasts. It would be reasonable to suggest a chimeric effect. However, although the two types of cells can be observed under the microscope, no distribution of sectors could be found with the naked eye (Fig. 1A), and these two types of cells can even be found distributed in a single juice vesicle (Fig. 4). These observations indicated that the fruit is not likely to be a chimera. Therefore the genetic background for each flesh cell should be same. Thus, comparison of the yellow and the red juice vesicles separated from Cara Cara flesh with modern –omics techniques can serve as an ideal starting point for further exploration of the mechanisms for high carotene accumulation.

## Methods

### Plant Materials

Fruits of Newhall (yellow-fleshed) and Cara Cara (red-fleshed) oranges (*Citrus sinensis* L. Osbeck) were sampled from an orchard in Linhai, Zhejiang, China. Fruits at eight different developmental stages, i.e., S1 to S8, from fruitlet to full mature (Fig. 1A) were collected from ten trees, with at least one representative fruit from each tree. The fruit were randomly separated into three biological replicates, with at least three fruit for each replicate. After color and size measurements of the fruit, the flesh tissue was separated and immediately frozen in liquid nitrogen, then stored at −70 °C for further analyses.

For allele expression analysis, juice vesicles from a single Cara Cara fruit at S6 were separated, with the most yellow ones (20% of the total) saved as yellow juice vesicles, and the 20% most red ones as red juice vesicles. Three fruit were involved with each as a biological replicate.

For NFZ and CPTA treatments, fruit were picked at S5 and were surface-sterilized as described^55^. Juice segments were excised from the equatorial region of the fruit, placed on Murashige and Skoog (MS) medium supplemented with 10% (w/v) sucrose and 1% (w/v) agar, pH = 5.8. The segments were placed with the endocarp side uppermost in the dark at 25 °C. CPTA and NFZ were added to the culture mediums to final concentrations of 0.2% and 0.1 mM, respectively. At 0, 2, 4 weeks after treatments, the segments were sampled, juice vesicles and albedo layer tissues separated, and stored at −70 °C for further analysis. For each sampling, nine segments were taken and separated into three biological replicates with three segments in each replicate.

### Color and fruit size measurements

Peel color was measured using a Hunter Lab Mini Scan XE Plus colorimeter (Hunter Associates Laboratory, Inc., USA). The CIE L*a*b*color scale was adopted, and the data were expressed as L*, a*, b*, C*, H°, and the citrus color index (CCI) using the formula CCI = 1000 × a*/(L* × b*). Four random measurements per fruit were made. Fruit size at different stages was measured with Vernier calipers, using the transverse diameter as the fruit size.

### Carotenoid extraction, quantification, and HPLC analysis

Carotenoids were extracted from flesh tissues, analysed and quantified by HPLC, according to a method previously described^28^. Briefly, around 0.5 g of fresh tissues were extracted with chloroform/methanol/Tris, and after centrifugation, the chloroform phase from two extractions were combined and dried under nitrogen gas. The residue was dissolved in diethyl ether and then saponified with 6% KOH. Water and chloroform were added to the saponified mixtures and after separation the chloroform phases were collected and dried under nitrogen gas. A Waters Alliance 2695 system (Waters Corporation, USA) equipped with a YMC reverse-phase C_30_ column was used for HPLC analysis.

### Cell squashing and differential interference contrast (DIC) microscopy

The flesh tissue was cut into small pieces with a sterile scalpel blade and fixed overnight in 2.5% glutaraldehyde in 0.1 M phosphate buffer (pH 7.0). Then the fixing solution was removed and 0.1 M Na_2_EDTA was added to the samples, and then incubated at 60 °C for 2.5 h^56^. The cells were separated by squashing and observed and photographed with a Zeiss microscope (Germany).

### Hyperspectral confocal Raman microscopy measurements

The Raman microscopy measurements were performed on a Renishaw inVia laser-scanning confocal Raman microscope. The laser emitted light at 532 nm and the laser beam power was 50 mW. The signals were collected with the following parameters: spectral resolution less than 1 cm^−1^, exposure time 1 s, confocality high. A single juice vesicle was used to make one freehand section. The testing laser beam was pointed precisely at a single chromoplast and each measurement determined separately using Raman microscopy^37^.

### Transmission electron microscopy (TEM)

Juice vesicles were separated and fixed overnight at 4 °C in 2.5% glutaraldehyde in 0.1 M phosphate buffer (pH 7.0). Samples were washed three times, 15 min for each, with phosphate buffer, then post-fixed with 1% OsO_4_ in 0.1 M phosphate buffer (pH 7.0) for 1–2 h and washed three times in phosphate buffer. Samples were dehydrated by a linear gradient ethanol series (50%, 70%, 80%, 90%, 95%, and 100%), 15 min for each, and infiltrated by absolute acetone for 20 min. Samples were infiltrated with resin gradually (1:1 mixture of absolute acetone and the final Spurr resin mixture for 1 h, a 1:3 mixture of absolute acetone and the final resin mixture for 3 h, and finally the Spurr resin mixture overnight). Embedded samples were placed in capsules contained embedding medium and heated at 70 °C for 9 h. The samples were stained with uranyl acetate and alkaline lead citrate for 15 min each and observed in TEM using a Hitachi JEM-1230 (Japan).

### Isolation of RNA and synthesis of cDNA

Total RNA was extracted from fruit frozen powder as previously described^22^. RNA integrity was electrophoretically verified and genomic DNA was eliminate by DNase I (RNase-free) (Fermentas MBI). The cDNA was synthesized using an iScript^TM^ cDNA Synthesis Kit (Bio-Rad, USA). All procedures were according to the instruction manual.

### Full-length gene cloning and sequencing

Primers for full-length cloning of *PSY1*, *LCYB* and *CYCB* were designed according to consensus sequences flanking coding sequences of each gene from *C. unshiu*, *C. paradisi* and *C. sinensis* deposited in GenBank and the sequences of primers are listed in Supplementary Table S3. PCR was performed using High Fidelity PCR Master kit (Roche, Switzerland) and general cloning procedures were followed. Sequences for both alleles were obtained from sequencing of at least ten recombinant plasmids for each gene.

### Quantitative real-time PCR analysis

The quantitative real-time reactions were performed with a CFX96 instrument (Bio-Rad, USA) in a 20 μl system including 1 μl of each primer (Supplementary Table S1), 2 μl cDNA, 6 μl PCR-grade water, and 10 μl SsoFast^TM^ EvaGreen Supermix (Bio-Rad, USA). The PCR programme was initiated with a preliminary step of 5 min at 95 °C, followed by 45 cycles at 95 °C for 5 s, 58 °C for 15 s, and 72 °C for 10 s. A melting curve was generated for each sample at the end of each run to assess the purity of the amplified products. The expression level of *actin* was used to normalize the mRNA levels for each sample, with abundance expressed as a multiple of *actin*. The expression level of each of the *PSY1* and *CYCB* allele was measured by quantitative real-time PCR using allele-specific PCR primers (Supplementary Table S1). All PCR products were cloned and sequenced to verify the authenticity of the amplification.

## Electronic supplementary material

Supplementary data
