# Supplementary material for: Cytological and molecular characterization of carotenoid accumulation in normal and high-lycopene mutant oranges
Source: Sci Rep. 2017 Apr 10;7:761. doi: 10.1038/s41598-017-00898-y (PMC5429694; doi:10.1038/s41598-017-00898-y)
Supplement: Supplementary file 1 — Supplementary data [file 41598_2017_898_MOESM1_ESM.pdf]

Cytological and molecular characterization of carotenoid accumulation in normal and high-lycopene mutant oranges

Peng-jun Lu, Chun-yan Wang, Ting-ting Yin, Si-lin Zhong, Don Grierson, Kun-song Chen & Chang-jie Xu

Supplementary data

Figure S1. Carotenoid biosynthetic pathway in higher plants with the sites inhibited by CPTA and NFZ indicated.

Figure S2. The mRNA sequences of *phytoene synthase 1* (*PSY1*) alleles, *PSY1-a* and *PSY1-b*, in Newhall and Cara Cara oranges.

Figure S3. The mRNA sequences of *lycopene β-cyclase* (*LCYB*) alleles, *LCYB-a* and *LCYB-b*, in Newhall and Cara Cara oranges.

Figure S4. The mRNA sequences of *chromoplast-specific lycopene β-cyclase* (*CYCB*) alleles, *CYCB-a* and *CYCB-b*, in Newhall and Cara Cara oranges.

Figure S5. The observation of red crystalline chromoplast under the light microscope in 2-(4-chlorophenylthio)-triethylamine hydrochloride (CPTA) treated albedo layers of Newhall, control Newhall and control Cara Cara oranges.

Table S1. List of primers for real-time quantitative PCR.

Table S2. Effects of 2-(4-chlorophenylthio)-triethylamine hydrochloride (CPTA) and norflurazon treatments on accumulation of carotenoids in *in vitro* cultured flesh tissues of Newhall and Cara Cara.

Table S3. List of primers for full-length gene cloning.

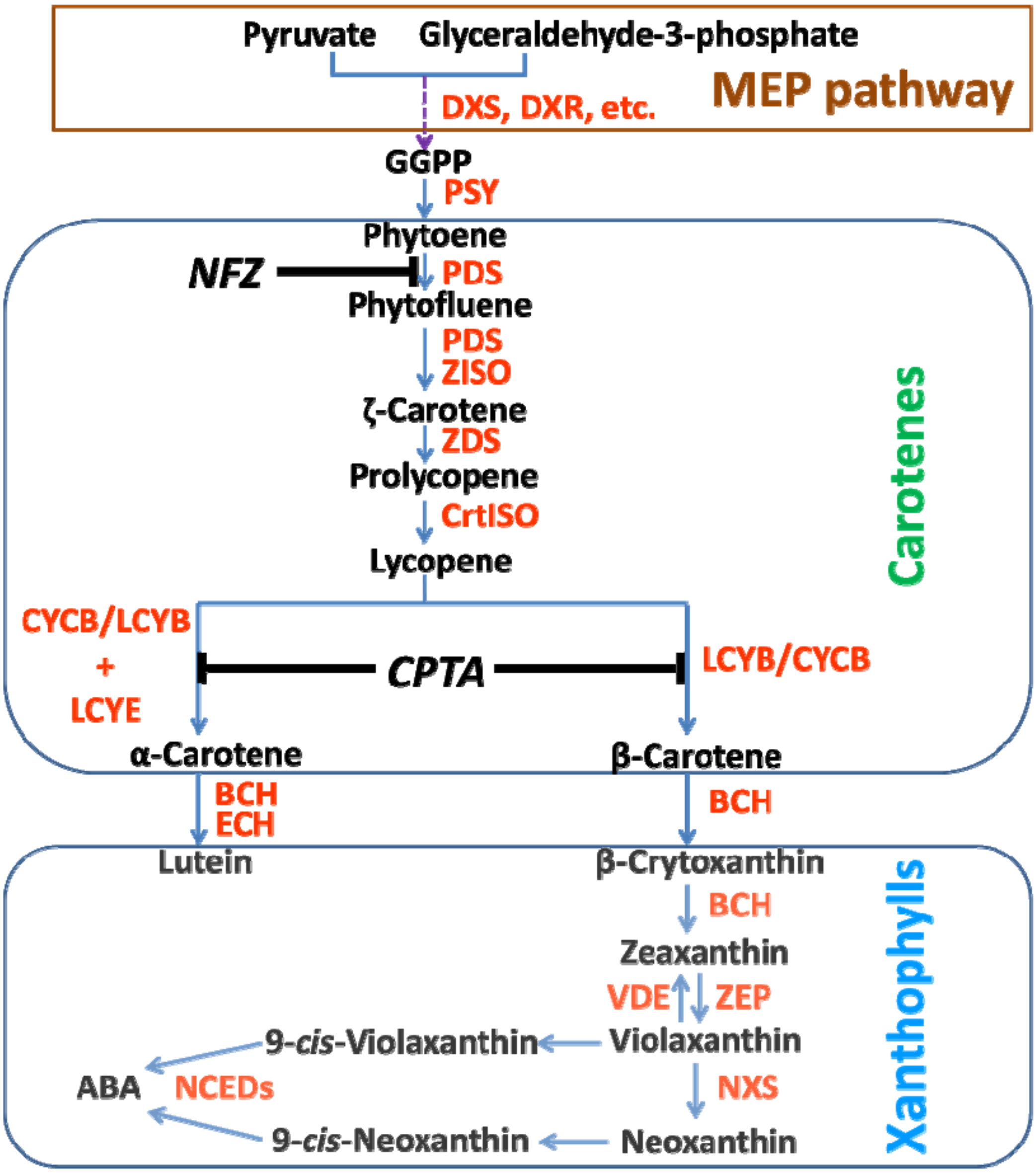

Supplementary Fig. S1. Carotenoid biosynthetic pathway in higher plants with the sites inhibited by CPTA and NFZ indicated. BCH, β-carotene hydroxylase; CPTA, 2-(4-chlorophenylthio)-triethylamine hydrochloride; CRTISO, carotene isomerase; CYCB, chromoplast-specific lycopene β-cyclase; DXS, 1-deoxy-D-xylulose 5-phosphate-synthase; DXR, 1-deoxy-D-xylulose 5-phosphate reductoisomerase; ECH, ε-carotene hydroxylase; GGPP, geranylgeranyl diphosphate; LCYB, lycopene β-cyclase; LCYE, lycopene ε-cyclase; MEP, 2-C-methyl-D-erythritol 4-phosphate; NCED, nine-*cis*-epoxycarotenoid dioxygenase; NFZ, norflurazon; NXS, neoxanthin synthase; PDS, phytoene desaturase; PSY, phytoene synthase; VDE, violaxanthin de-epoxidase; ZDS, ζ-carotene desaturase; ZEP, zeaxanthin epoxidase; ZISO, 15-*cis*-ζ-carotene isomerase

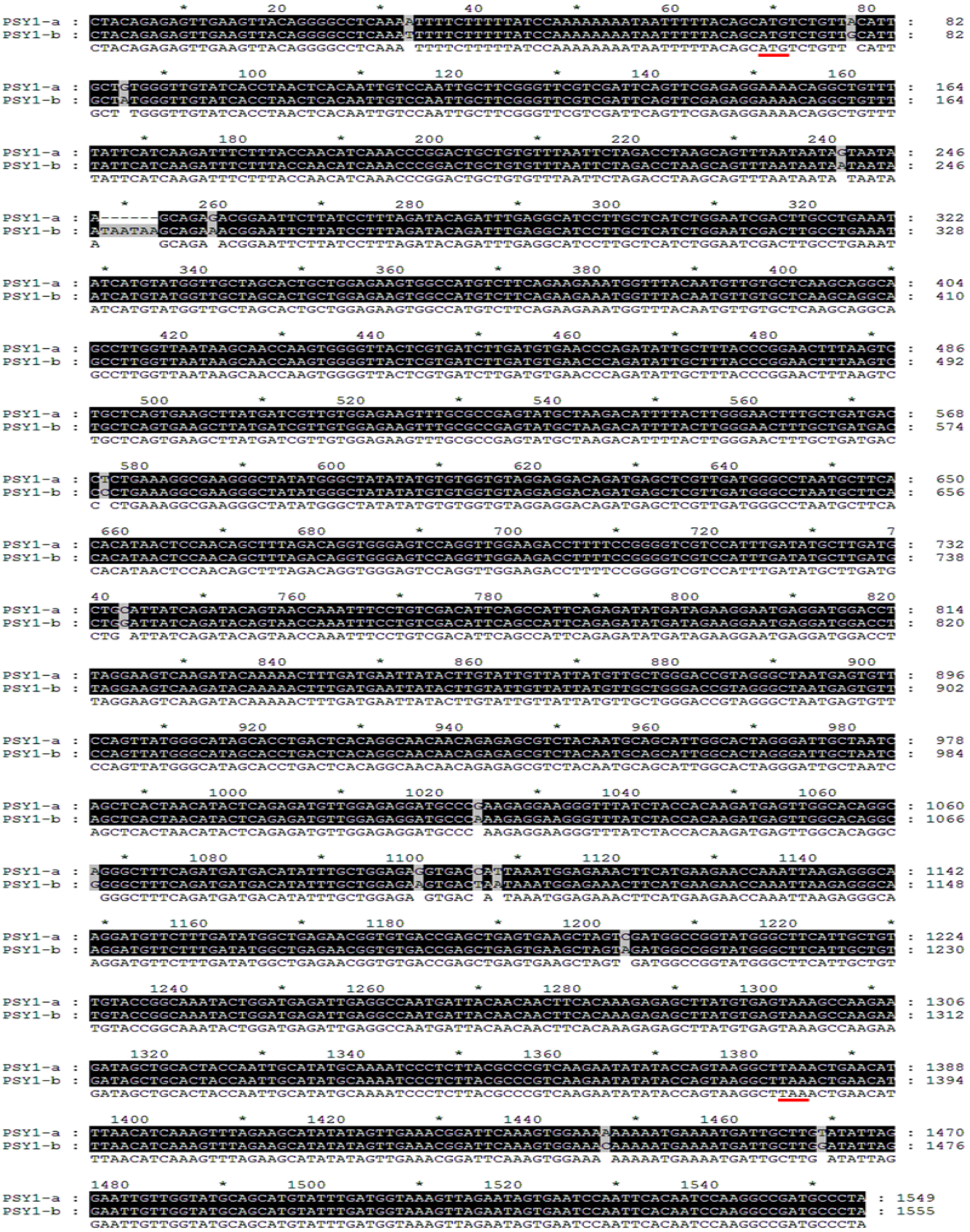

Supplementary Fig. S2. The mRNA sequences of *phytoene synthase 1* (*PSY1*) alleles, *PSY1-a* and *PSY1-b*, in Newhall and Cara Cara oranges. Note that, no difference in sequence was observed between the two oranges. Start codon and stop codon are underlined by red.

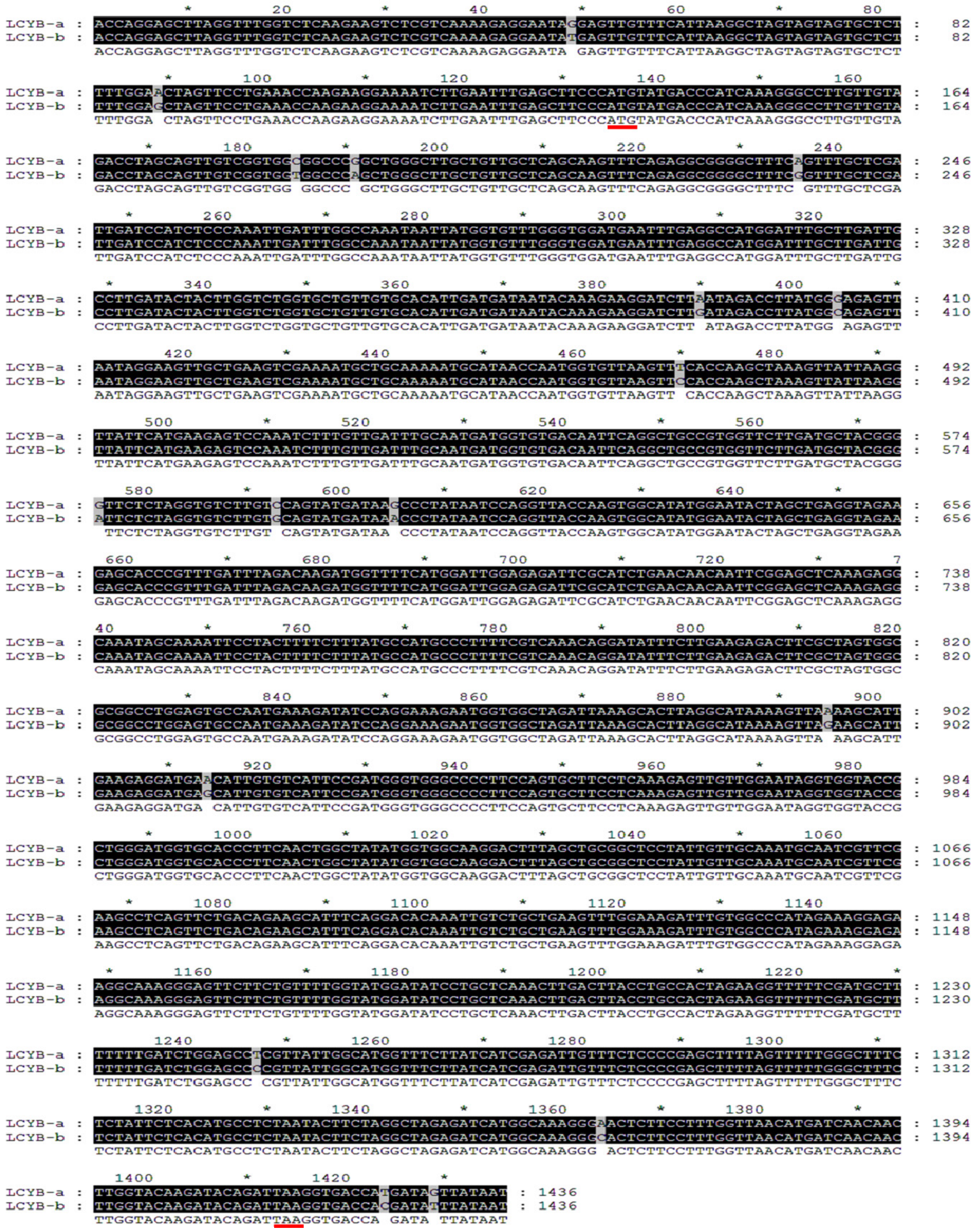

Supplementary Fig. S3. The mRNA sequences of *lycopene β-cyclase* (*LCYB*) alleles, *LCYB-a* and *LCYB-b*, in Newhall and Cara Cara oranges. Note that, no difference in sequence was observed between the two oranges. Start codon and stop codon are underlined by red.

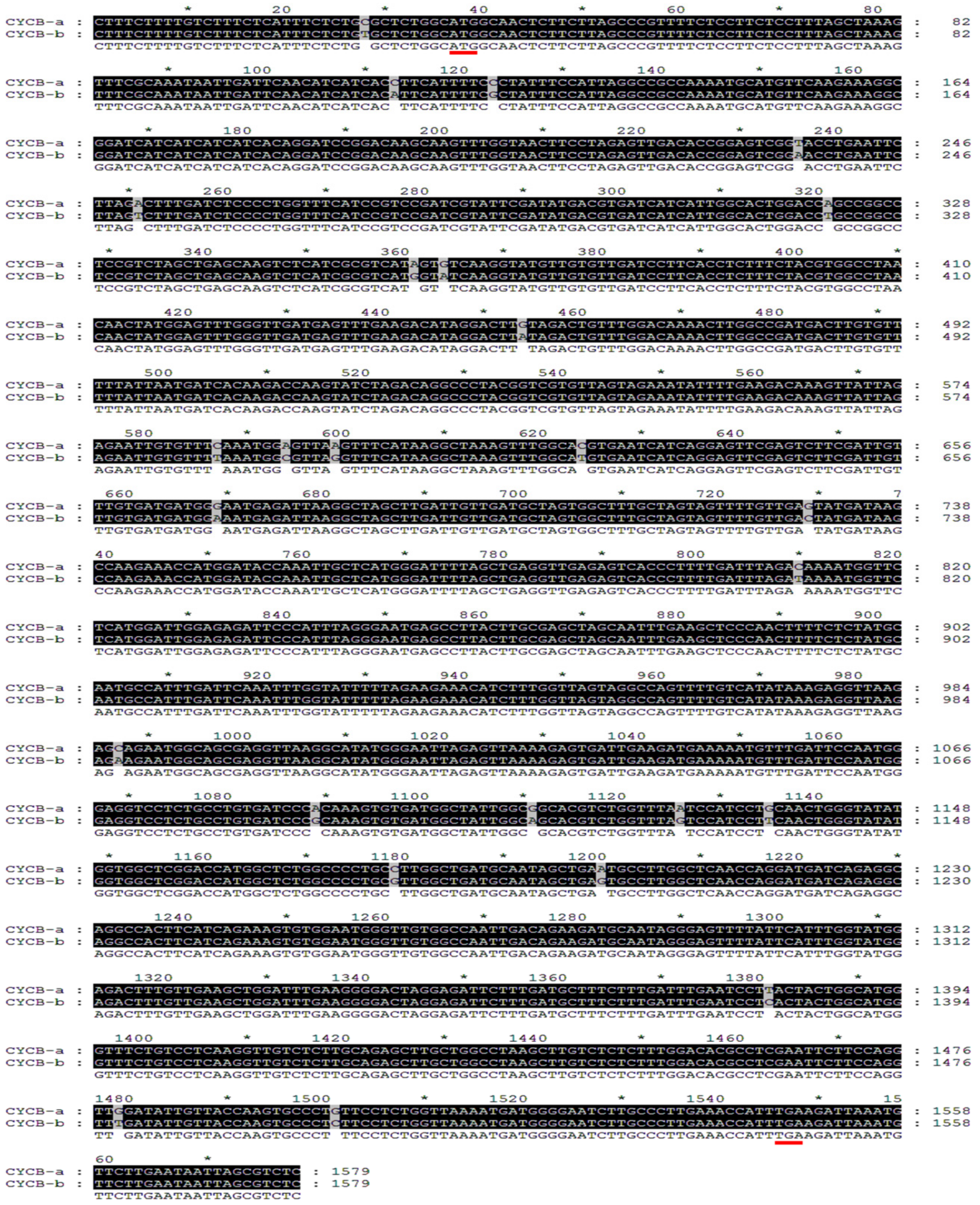

Supplementary Fig. S4. The mRNA sequences of *chromoplast-specific lycopene β-cyclase* (*CYCB*) alleles, *CYCB-a* and *CYCB-b*, in Newhall and Cara Cara oranges. Note that, no difference in sequence was observed between the two oranges. Start codon and stop codon are underlined by red.

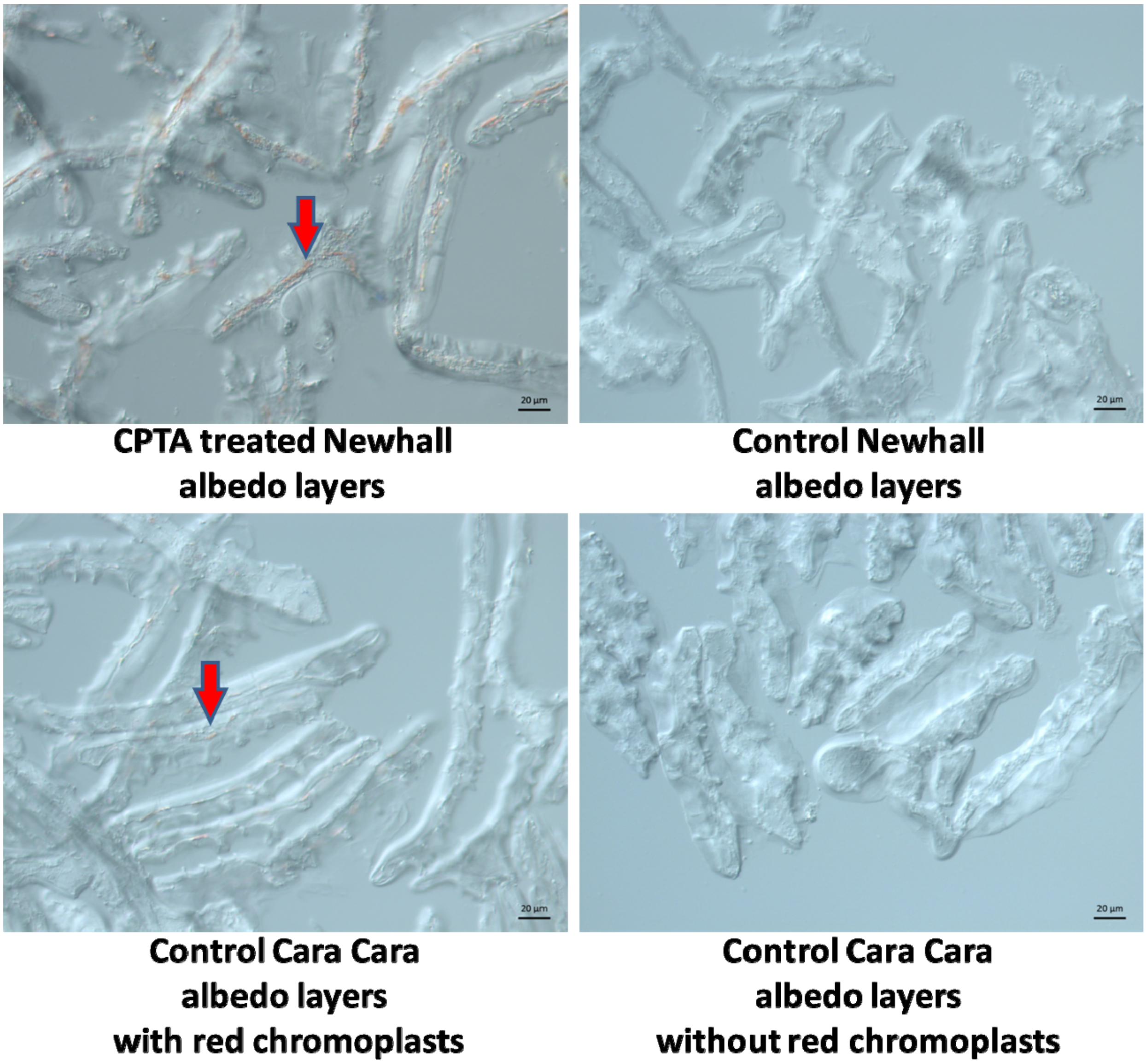

Supplementary Fig. S5. The observation of red crystalline chromoplast under the light microscope in 2-(4-chlorophenylthio)-triethylamine hydrochloride (CPTA) treated albedo layers of Newhall, control Newhall and control Cara Cara oranges. Bar, 20μm.

Supplementary Table S1. List of primers for real-time quantitative PCR

Gene	Primers (Forward; Reverse)	Genome accession
Actin	5'-CATCCCTCAGCACCTTCC-3';<br>5'-CCAACCTTAGCACTTCTCC-3'	Cs1g05000
BCH	5'-GTTTGCCATAATCAACGC-3';<br>5'-CTCTCCGGAAATAAGGCA-3'	Cs9g19270
CCD1	5'-GCACGAAATGGAATAGTGGC-3';<br>5'-CTTTAGTAGGAGGAGTCTCG-3'	Cs7g01710
CRTISO	5'-ATTTGATAACCCAAGCACTG-3';<br>5'-TGAGGCAGATAATAGGCAAG-3'	Cs6g13340
CYCB	5'-CCCTATTTCCATTAGGCCGC-3';<br>5'-CACGTCATATCGAATACGATC-3'	Cs1g04120
CYCB-a	5'-GAGCAAGTCTCATCGCGTCATA-3'<br>5'-ACTTTAGCCTTATGAAACTTAACTCCATTTG-3'	
CYCB-b	5'-GCAAGTCTCATCGCGTCATGGTA-3'<br>5'-ACTTTAGC CTTATGAAACCTAACGCCATTTA-3	
DXR	5'-GAAGTTAAAGTGGCTGATGC-3';<br>5'-GAAGAATCCTGTGTTTCGAC-3'	Cs5g05440
DXS1	5'-CCGCATTCTCATTCCCGACT-3';<br>5'-TTGTGAGTGATACTCTCCTCT-3'	Cs9g05150
DXS2	5'-TTCCGGGATTGCTATTGGAG-3';<br>5'- AAATCAATTTTCCAGCCGCC-3'	Cs1g20530
LCYB	5'-GAACCAGGAGCTTAGGTCTG-3';<br>5'-GCTAGGTCTACAACAAGGCC-3'	orange1.1t00772
LCYE	5'-AACCCATCTTGATTGGTCGT-3';<br>5'-AGAAGCAACAGTAGCAAGCCT-3'	Cs4g14850
NCED2	5'-CCATGGCCTTGGACATGGCG-3';<br>5'-TGATGCATTCGGGGATGGAG-3'	Cs2g03270
NCED3	5'-CTTTAGCCTTGGACGCAGTC-3';<br>5'-GAATGCAGTCGGGGACCTTT-3'	Cs5g14370
PDS	5'-TTCAGCCGATTTGATTTTCC-3';<br>5'-ACACCCTGCTTTCTCATCCA-3'	orange1.1t02361
PSY1	5'-CTACAATGCAGCATTGGCAC-3';<br>5'-CATGAAGTTTCTCCATTTAATGG-3'	Cs6g15910
PSY1-a	5'-CAAGATGAGTTGGCACAGGCA-3'<br>5'-AGCCCATACCGGCCATCG-3'	
PSY1-b	5'-CAAGATGAGTTGGCACAGGCG-3'<br>5'-GAAGCCCATACCGGCCATCT-3'	
PSY2	5'-TTACAATGCAGCATTGGCCT-3';<br>5'-CATGAAGTTTCTCCATTTATTAAT-3'	orange1.1t00657
VDE	5'-ACCGAATGCCAGATCAAATGTGG-3';<br>5'-CCACCATATCCATCCCATGC(A/G)TC-3'	Cs5g26080
ZDS	5'-TCATCCCAAGGTTTAGAAG-3';<br>5'-TACCAGACAAAGTTGCTCC-3'	orange1.1t06069
ZEP	5'-ACTTGTTACACTGGAATTGCAGA-3';<br>5'-CCCATATTTGGCTGCATAGCATG-3'	orange1.1t04051
ZISO	5'-ACTCCGTCTCCTCCCTTTCA-3';<br>5'-TTACCTGCCCAACCATCTGT-3'	Cs5g24730

Supplementary Table S2. Effects of 2-(4-chlorophenylthio)-triethylamine hydrochloride (CPTA) and norflurazon treatments on accumulation of carotenoids in *in vitro* cultured flesh tissues of Newhall and Cara Cara. The 0 week tissues were separated from fruit at S5 stage.

Treatment	Contents (μg g^{-1} FW)								
	Phytoene	Phytofluene	β-Carotene	Lycopene	Violaxanthin	Luteoxanthin	*cis*-Violaxanthin	Others	Total carotenoids
Control (2 weeks) Newhall					0.20 ±0.05	0.20 ±0.02	0.56 ±0.11	0.12 ±0.02	1.08 ±0.13
Control (2 weeks) Cara Cara	24.07 ±0.20	3.87 ±0.17	0.14 ±0.08	5.85 ±0.30	0.12 ±0.03	0.11 ±0.02	0.36 ±0.05	0.57 ±0.05	35.08 ±0.70
CPTA (2 weeks) Newhall	1.60 ±0.07	0.39 ±0.03		13.91 ±0.89			0.14 ±0.01	1.41 ±0.11	17.44 ±1.02
CPTA (2 weeks) Cara Cara	10.64 ±0.49	1.91 ±0.11	0.24 ±0.03	7.39 ±0.45			0.03 ±0.02	0.82 ±0.01	21.04 ±0.98
Norflurazon (2 weeks) Newhall	32.22 ± 0.53	1.77 ±0.03			0.01 ± 0.00	0.03 ± 0.00	0.04 ± 0.00		34.07 ±0.55
Norflurazon (2 weeks) Cara Cara	46.60 ±1.34	3.04 ±0.08		2.62 ±0.17			0.02 ±0.01	0.53 ±0.03	52.81 ±1.48
Control (4 weeks) Newhall	0.03 ±0.03				0.17 ±0.01	0.27 ±0.02	0.60 ±0.05	0.41 ±0.03	1.48 ±0.04
Control (4 weeks) Cara Cara	33.44 ±1.44	5.33 ±0.24		5.37 ±0.46	0.10 ±0.02	0.19 ±0.05	0.37 ±0.04	0.85 ±0.09	45.64 ±2.24
CPTA (4 weeks) Newhall	2.52 ±0.03	0.83 ±0.02		18.13 ±0.69	0.05 ±0.00	0.03 ±0.00	0.24 ±0.00	1.40 ±0.06	23.20 ±0.80
CPTA (4 weeks) Cara Cara	31.48 ±1.28	6.25 ±0.23		16.64 ±0.74	0.02 ±0.00		0.08 ±0.02	1.47 ±0.05	55.95 ±2.29
Norflurazon (4 weeks) Newhall	60.09 ±3.37	4.13 ±0.20			0.04 ±0.01	0.07 ±0.00	0.13 ±0.02	0.08 ±0.01	64.54 ±3.59
Norflurazon (4 weeks) Cara Cara	82.76 ±2.15	5.10 ±0.17		0.64 ±0.00		0.03 ±0.00	0.05 ±0.02	0.24 ±0.04	88.82 ±2.31

Supplementary Table S3. List of primers for full-length gene cloning

Gene	Primers (Forward; Reverse)
CYCB	5’- CTTTCTTTTGTCTTTCTCATTTC-3’
	5’- GAGACGCTAATTATTCAAGAAC-3’
LCYB	5’-TGAAGATTCAGAACCAGGAG-3’
	5’- GCATGAGTTATTAAGCACATTA-3’
PSY1	5’-CTACAGAGAGTTGAAGTTACAGGG-3’
	5’-TAGGGCATCGGCCTTGGATTGTG-3’
